# Evaluation and comparison the performance of titanium and zirconium(IV) tetrachloride in textile wastewater treatment

**DOI:** 10.1016/j.dib.2018.03.113

**Published:** 2018-03-28

**Authors:** Hamidi Abdul Aziz, Muhamad Haziq Abd Razak, Muhammad Zamir Abdul Rahim, Wan Izatul Saadiah Wan Kamar, Salem S. Abu Amr, Sabir Hussain, John Van Leeuwen

**Affiliations:** a School of Civil Engineering, Engineering Campus, Universiti Sains Malaysia, 14300 NibongTebal, Penang, Malaysia; b Solid Waste Management Cluster, Science and Engineering Research Center, Engineering Campus, Universiti Sains Malaysia, 14300 Nibong Tebal, Penang, Malaysia; c Malaysian Institute of Chemical and Bioengineering Technology, Universiti Kuala Lumpur (UniKl, MICET), Melaka, Malaysia; d Natural & Built Environments Research Centre, School of Natural and Built Environments, University of South Australia, South Australia 5095, Australia

**Keywords:** Textile wastewater, Coagulation, Titanium tetrachloride, Zirconium tetrachloride

## Abstract

Wastewater treatment is a key challenge in the textile industry. The current treatment methods for textile wastewater are insufficient or ineffective for complex dyes generated from the textile industry. This study evaluated the performances of two novel inorganic coagulants with high cationic charges, namely, titanium tetrachloride (TiCl_4_) and zirconium tetrachloride (ZrCl_4_). They were utilised to treat textile industry wastewater. Both coagulation processes were performed under the same experimental operational conditions. Turbidity, suspended solids (SS), colour, chemical oxygen demand (COD) and ammonia were measured to assess the efficiencies of the coagulants. Results indicated that ZrCl_4_ and TiCl_4_ exhibited high potentials for textile wastewater treatment. ZrCl_4_ presented high removal efficiency in COD and SS, whereas TiCl_4_ showed excellent removal in ammonia.

**Specifications Table**

**Table:** 

**Subject area**	Environmental Engineering
**More specific subject area**	Wastewater treatment
**Type of data**	Tables and figures
**How data was acquired**	All experiments were performed in 1000 mL glass beakers using jar test unit. ZrCl4 and TiCl4 were used as coagulants to treat textile wastewater samples. COD concentration, TSS, colour and ammonia were measured before and after each run, and the removal efficiencies were calculated.
**Data format**	Analysed
**Experimental factor**	Monitoring the removal efficiencies of turbidity, suspended solids, colour, COD, and ammonia from textile wastewater after each coagulation process.
**Experimental features**	Treatment of textile wastewater using ZrCl_4_ and TiCl_4_ as a coagulation process, and compare the performance of both coagulants based on the maximum removal efficiencies for each parameter.
**Data source location**	School of Civil Engineering, Engineering Campus, Universiti Sains Malaysia, 14300 NibongTebal, Penang, Malaysia
**Data accessibility**	Data were presented in the article.

**Value of the data**

• This article presents the data on the performances of two coagulants, ZrCl4 and TiCl4, in textile wastewater treatment
• The focus was on the comparison of the removal efficiencies of the parameters in textile wastewater, such as COD, TSS, colour and ammonia, under the effects of both coagulants.
• The dataset could also be used for reducing other parameters from other types of industrial wastewater, which is a challenging pollutant of natural water bodies.

## 1 Data

**Fig. 1:**
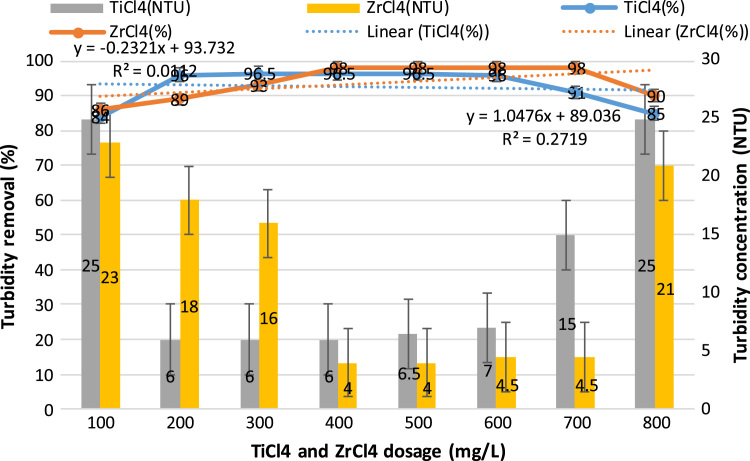
Actual (NTU) and percentage turbidity removals using ZrCl4 and TiCl4 as coagulants.

**Fig. 2:**
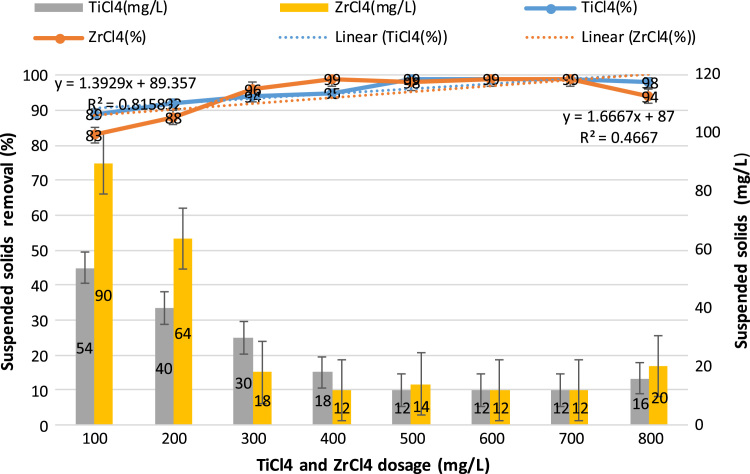
Percentage SS removals by ZrCl4 and TiCl4 as coagulants and SS concentrations after treatments.

**Fig. 3:**
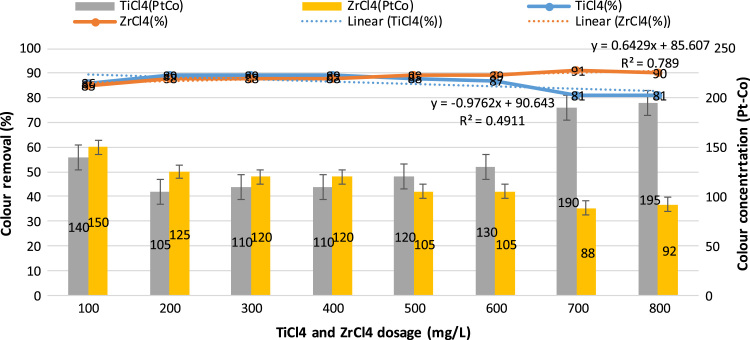
Percentage removals of colour by ZrCl4 and TiCl4 coagulants and their residual (PtCo) after treatment.

**Fig. 4:**
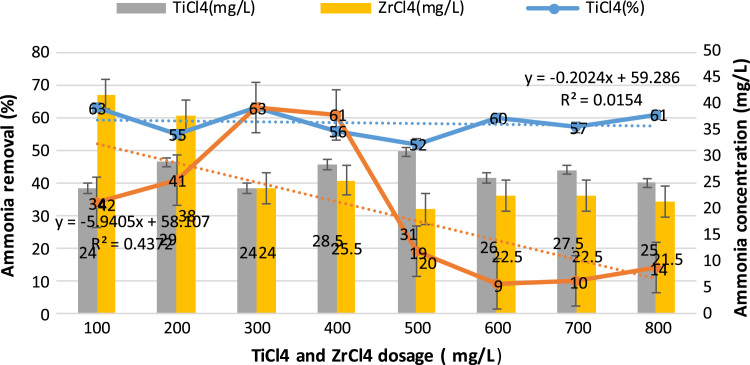
Percentage removal of ammonia for ZrCl_4_ and TiCl_4_ as coagulant in textile wastewater treatment.

**Fig. 5:**
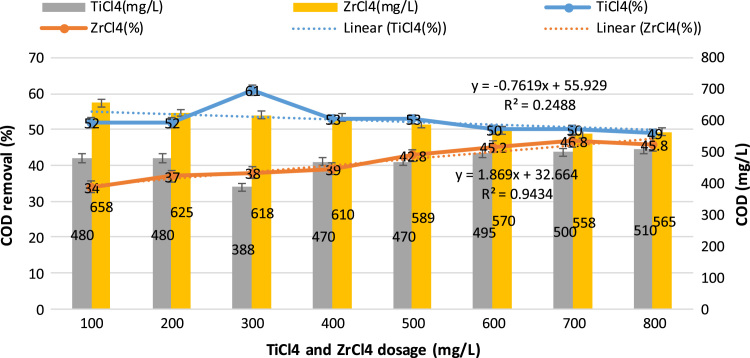
Percentage removals of COD by ZrCl4 and TiCl4 as coagulants in textile wastewater.

**Fig. 6:**
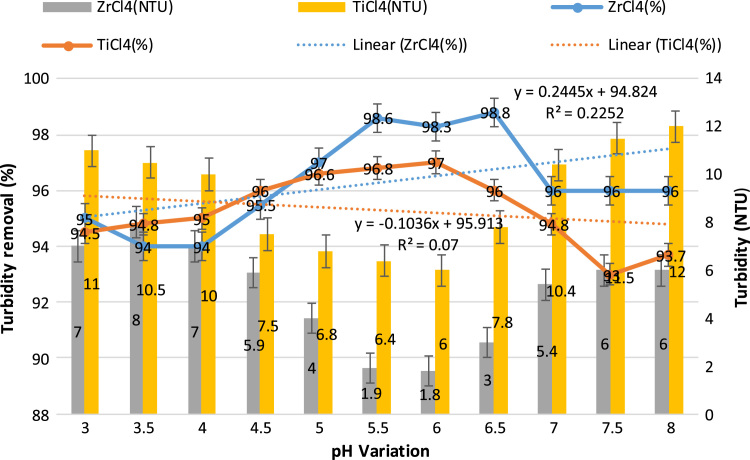
Turbidity results of the ZrCl4 and TiCl4 treatments of textile wastewater over the 3–8 pH range.

**Fig. 7:**
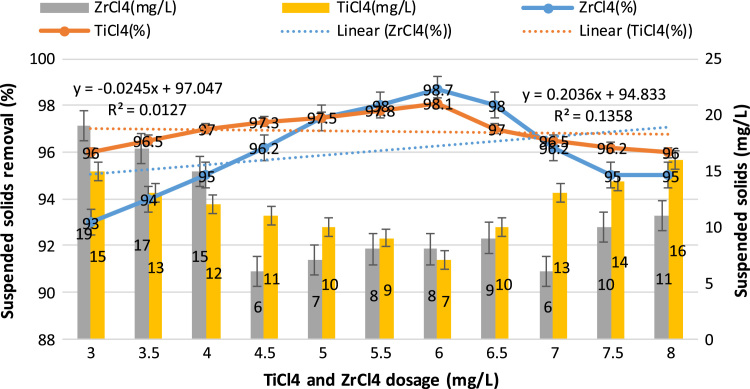
Removal of SS by ZrCl4 and TiCl4 as coagulants in textile wastewater treatment.

**Fig. 8:**
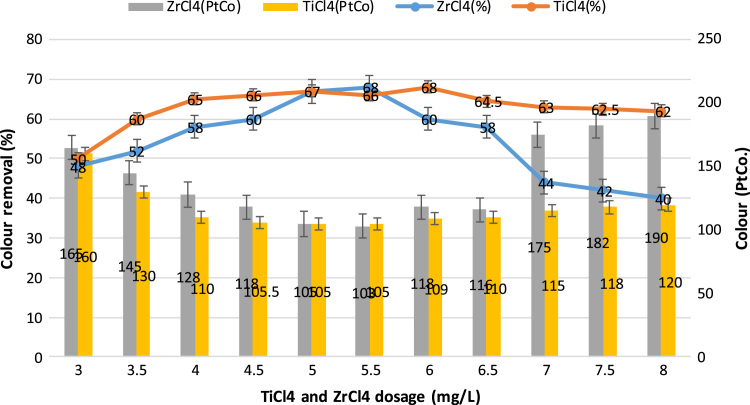
Removal of colour by ZrCl4 and TiCl4 as coagulants in textile wastewater treatment.

**Fig. 9:**
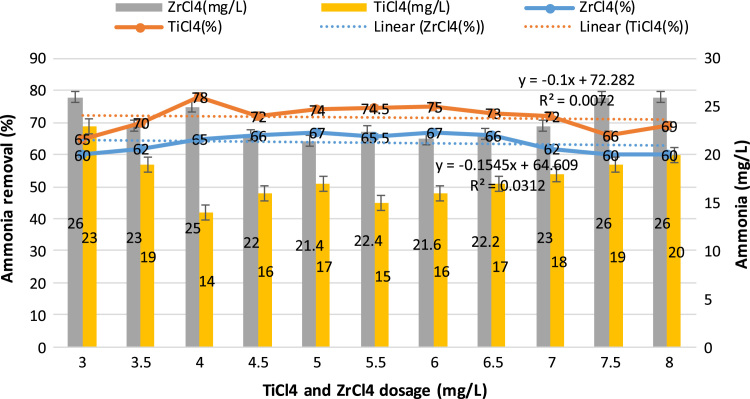
Removal of ammonia by ZrCl4 and TiCl4 as coagulants in textile wastewater treatment.

**Fig. 10:**
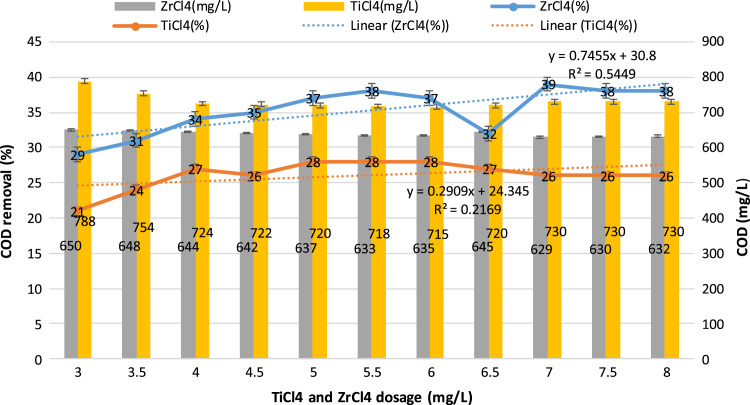
COD removal by ZrCl4 and TiCl4 as coagulants in textile wastewater treatment.

This study aimed to evaluate and compare the performances of titanium tetrachloride (TiCl_4_) and zirconium tetrachloride (ZrCl_4_) as coagulants in textile wastewater treatment. The performances of both coagulants were compared through the removal of such parameters as turbidity, suspended solids (SS), ammonia, chemical oxygen demand (COD) and colour. Table 1 presents the general characteristics of textile wastewater compared with Standard B of Environmental Quality (Sewage and Industrial Effluents) Regulation 2012 under the Environmental Quality Act 2012. Fig. 1, Fig. 2, Fig. 3, Fig. 4, Fig. 5 show the performances of ZrCl_4_ and TiCl_4_ in removing turbidity, SS, colour, ammonia and COD, respectively, under the effects of different coagulant dosages at natural wastewater pH. Fig. 5, Fig. 6, Fig. 7, Fig. 8, Fig. 9, Fig. 10 present the effect of pH variation on the performances of the two coagulants (ZrCl_4_ and TiCl_4_) for turbidity, SS, colour, ammonia and COD removals. Table 2 summarise the operational conditions and performances of ZrCl4 and TiCl4 in parameter removal.

**Table 1:** Characteristics of textile wastewater compared with Standard B of Environmental Quality (Sewage and Industrial Effluents) Regulation 2012 under the Environmental Quality Act 2012.

Parameter	Textile wastewater		Standard B
	Untreated^1^	Treated^2^	
pH	11.8	7.9	5.5 – 9.0
COD (mg/L)	998	552	250
Turbidity (NTU)	159	2.31	–
Colour (Pt.Co)	1020	860	–
Suspended Solid (mg/L)	540	40	100
Ammonia (mg/L)	65	8.2	–
BOD_5_ (mg/L)	80	24	40

**Table 2:** Summary of the comparison of the performances of ZrCl_4_ and TiCl_4_ as coagulants.

Item	ZrCl_4_	TiCl_4_
**Operating condition** pH	6	6
Coagulant dose(mg/L)	500	300
Rapid mixing time(min)	1	1
Rapid mixing speed(rpm)	250	250
Slow mixing time(min)	20	20
Slow mixing speed(rpm)	30	30
Settling time (min)	30	30
		
**Removal rate (%):**		
i)Turbidity	97	97
ii)Colour	60	68
iii)Suspended solids	99	98
iv)COD	36	28
v)Ammonia	67	28

## 2 Experimental design, materials and methods

### 2.1 Sampling

A textile wastewater sample was collected from a textile factory in Prai Industrial Estate, Penang, was immediately transferred to the laboratory and was stored at 4 °C, in accordance with the Standard Methods for the Examination of Water and Wastewater [1]. The parameters measured for assessing the coagulants were pH, colour, turbidity, SS, COD, biochemical oxygen demand, ammonia, alkalinity and hardness.

### 2.2 Preparation of stock solution

Stock solutions of both coagulants were prepared before each experiment. The TiCl_4_ stock solution was prepared by adding 1 mL of TiCl_4_ to 0.2 M hydrochloric acid. A 5000 mg/L stock solution of ZrCl_4_ was prepared by dissolving 5 g of ZrCl4 in 1000 mL of distilled water. These stock solutions were stored for approximately 14 h at room temperature to dissolve the metal salts [2].

### 2.3 Coagulation-flocculation

Jar testing was conducted under standardised conditions on wastewater to evaluate coagulant dosages and conditions required to achieve the optimum treatment process. A conventional jar test apparatus was used in experiments to evaluate the performances of ZrCl_4_ and TiCl_4_ in treating textile wastewater. An automated jar test apparatus, which consisted of six 1000 mL beakers and six-spindle steel paddles, was used. The jar testing was performed after leaving the textile samples at ambient temperature for 2 h. Stock solutions of ZrCl_4_ and TiCl_4_ coagulants were prepared before each experiment. The sample of textile wastewater was mixed with the coagulants before being poured into 500 mL beakers. After coagulant dosing into the wastewater samples, rapid mixing at 250 rpm for 2 min and slow mixing at 30 rpm for 20 min were performed. Flocs were allowed to settle for 30 min [2], [3]. The treated wastewater samples were collected using a syringe from the supernatant surface for parameter measurements. In the jar test experiments, the TiCl_4_ doses were varied from 100 mg/L to 800 mg/L at pH 3 to pH 8. The ZrCl_4_ doses were varied from 300 mg/L to 800 mg/L at pH 4.5 to pH 7. The turbidity, colour, ammonia, COD and SS before and after treatment were measured. pH was measured on-site by using a portable digital pH/mV meter (WITEG, W-100, Germany) [4]. Colour measurements were reported as true colour (filtered using a 0.45 µm filter paper) at 455 nm using DR 2800 HACH spectrophotometer in accordance with the Standard Methods for the Examination of Water and Wastewater [1] (Method No. 2120 C). The result was reported in platinum–cobalt (PtCo) which was the unit of colour being produced by 1 mg platinum/L in the form of chloroplatinate ion. Turbidity was determined using a DR/2100 turbiditimeter. SS was measured using a DR2800 spectrophotometer in accordance with the HACH standard: Photometric Method 8006. Ammonia was determined using a DR2800 spectrophotometer in accordance with the Nessler method [5], adopted from the Standard Methods for the Examination of Water and Wastewater 4500-NH_3_ B and C. COD was determined in accordance with Method 5220D (closed reflux, colourimetric method) [5]. The removal efficiencies of turbidity, SS, colour, ammonia and COD were obtained using the following equation:

(1) Removal(%)=[(Ci−Cf)Ci]100

Where Ci and Cf are the initial and final concentrations of leachate, respectively.
